# *Fuz* Regulates Craniofacial Development through Tissue Specific Responses to Signaling Factors

**DOI:** 10.1371/journal.pone.0024608

**Published:** 2011-09-14

**Authors:** Zichao Zhang, Bogdan J. Wlodarczyk, Karen Niederreither, Shankar Venugopalan, Sergio Florez, Richard H. Finnell, Brad A. Amendt

**Affiliations:** 1 Institute of Biosciences and Technology, Texas A&M Health Science Center, Houston, Texas, United States of America; 2 Dell Pediatric Research Institute, University of Texas, Austin, Texas, United States of America; Massachusetts General Hospital, United States of America

## Abstract

The planar cell polarity effector gene *Fuz* regulates ciliogenesis and *Fuz* loss of function studies reveal an array of embryonic phenotypes. However, cilia defects can affect many signaling pathways and, in humans, cilia defects underlie several craniofacial anomalies. To address this, we analyzed the craniofacial phenotype and signaling responses of the *Fuz^−/−^* mice. We demonstrate a unique role for *Fuz* in regulating both Hedgehog (Hh) and Wnt/β-catenin signaling during craniofacial development. *Fuz* expression first appears in the dorsal tissues and later in ventral tissues and craniofacial regions during embryonic development coincident with cilia development. The *Fuz^−/−^* mice exhibit severe craniofacial deformities including anophthalmia, agenesis of the tongue and incisors, a hypoplastic mandible, cleft palate, ossification/skeletal defects and hyperplastic malformed Meckel's cartilage. Hh signaling is down-regulated in the *Fuz* null mice, while canonical Wnt signaling is up-regulated revealing the antagonistic relationship of these two pathways. Meckel's cartilage is expanded in the *Fuz^−/−^* mice due to increased cell proliferation associated with the up-regulation of Wnt canonical target genes and decreased non-canonical pathway genes. Interestingly, cilia development was decreased in the mandible mesenchyme of *Fuz* null mice, suggesting that cilia may antagonize Wnt signaling in this tissue. Furthermore, expression of *Fuz* decreased expression of Wnt pathway genes as well as a Wnt-dependent reporter. Finally, chromatin IP experiments demonstrate that β-catenin/TCF-binding directly regulates *Fuz* expression. These data demonstrate a new model for coordination of Hh and Wnt signaling and reveal a *Fuz*-dependent negative feedback loop controlling Wnt/β-catenin signaling.

## Introduction

Vertebrate craniofacial development is a complicated process requiring the coordination of multiple signaling pathways and tissue interactions among the three germ layers and neural crest cells in three dimensions. A number of signaling pathways have been implicated in craniofacial development including Hedgehog (Hh), Wnt/β-catenin, TGF-β, Fibroblast Growth Factor (Fgf), Notch, and Planar Cell Polarity (PCP) signaling [1], [2], [3], [4], [5]. However, the interactions and components shared among different signaling pathways are not well understood. The recent identification of the PCP effector gene *Fuzzy (Fuz)* as an important regulator of Hedgehog (Hh) signaling suggests that there may be substantial crosstalk between the different molecular cues. Furthermore, *Fuz* can coordinate ciliogenesis and secretion, two processes that affect a variety of signaling pathways [6], [7]. Our data suggest *Fuz* plays a pivotal role in the Wnt and Hedgehog pathways [6], [7], [8]. Furthermore, loss of *Fuz* leads to dramatic defects in craniofacial development.

PCP signaling, initially discovered in *Drosophila*, controls a diverse range of polarized cellular behaviors [9]. Recent studies have shown that PCP signaling is also important in regulating cell interactions and tissue movements [10], [11], [12]. In mice, loss of PCP genes leads to disruption of polarized structures such as the stereociliary bundles in the cochlea [13], [14], [15], [16]. In addition, mutants in PCP genes display an array of morphogenetic defects affecting the neural tube, heart, kidney and other tissues [1]. Thus, the PCP pathway affects a wide range of cell-cell interactions via coordination of morphogenesis, tissue polarity, and potentially growth.

The PCP pathway makes use of the Wnt signaling components Frizzled and Dishevelled, but is β-catenin-independent [17]. Studies of vertebrate planar cell polarity have primarily focused on the “core PCP” pathway. Thus, the function of vertebrate PCP “effectors”, which act downstream of Dishevelled, is unclear. This report focuses on one of the PCP effector proteins, Fuzzy or Fuz, which encodes a transmembrane protein required for tissue polarity. Elegant genetic studies in *Drosophila* have shown that *fuzzy*, along with *inturned*, plays a role in maintaining cytoskeletal integrity in the wing hairs; this role is genetically downstream of *dishevelled* and *frizzled* [18], [19], [20], [21].

Recent studies suggest that mutations affecting ciliogenesis lead to congenital human anomalies. Cilia appear to act as mechanical and chemical sensors that interpret extracellular cues via signaling pathways such as Hh, PDGF and Wnt [22]. Because there is no protein synthesis in the cilium, assembly and maintenance of the cilium requires intraflagellar transport (IFT). Thus, loss of IFT proteins leads to catastrophic effects on ciliogenesis and cilia function. Human conditions resulting from defects in ciliogenesis or IFT include situs inversus, polycystic kidney disease, ear defects and craniofacial anomalies [23], [24].

Several proteins involved in PCP signaling, including Dishevelled, localize to the base of cilia [25], [26], [27], [28]. This suggests that PCP signaling may be important for cilia function and possibly for coordination of other signaling pathways. In this context, we were particularly interested in the canonical Wnt/β-catenin pathway, as the absence of cilia results in enhanced β-catenin nuclear localization and downstream gene transcription [29], [30], [31].

In this study, we analyzed the expression, regulation and functional requirements of *Fuz* during vertebrate craniofacial development. We found that *Fuz^−/−^* mice display massive craniofacial defects including anophthalmia, agenesis of the tongue and incisors and a hypoplastic mandible leading to cleft palate. In contrast, we found that Meckel's cartilage, an embryonic structure that develops into the mandible and portions of the inner ear, was hyperplastic and malformed. Some aspects of the craniofacial phenotype, such as missing teeth and cleft palate, could be attributed to decreased Hh signaling. However, the increased growth of Meckel's cartilage was associated with increased Wnt signaling.

To further examine the molecular consequences of *Fuz* loss of function, we analyzed expression of Wnt target genes in *Fuz^−/−^* mice. We found that a number of β-catenin/TCF target genes were up-regulated in our mutants, suggesting that Fuz plays a dual role in Wnt signaling, in both the canonical and non-canonical pathways. Furthermore, we found that the Fuz protein could repress expression of Wnt pathway genes as well as a Wnt-dependent reporter, suggesting a direct role within the canonical pathway. Finally, most interesting, we identified β-catenin/TCF-binding sites in the *Fuz* gene, which we confirmed by chromatin immunoprecipitation. Together, these data imply that Fuz may be a critical factor linking the Hh, PCP and Wnt/β-catenin signaling pathways and may function as a switch to balance the activities of these pathways during craniofacial development.

## Results

### *Fuz* expression pattern during mouse development

**Figure 1:**
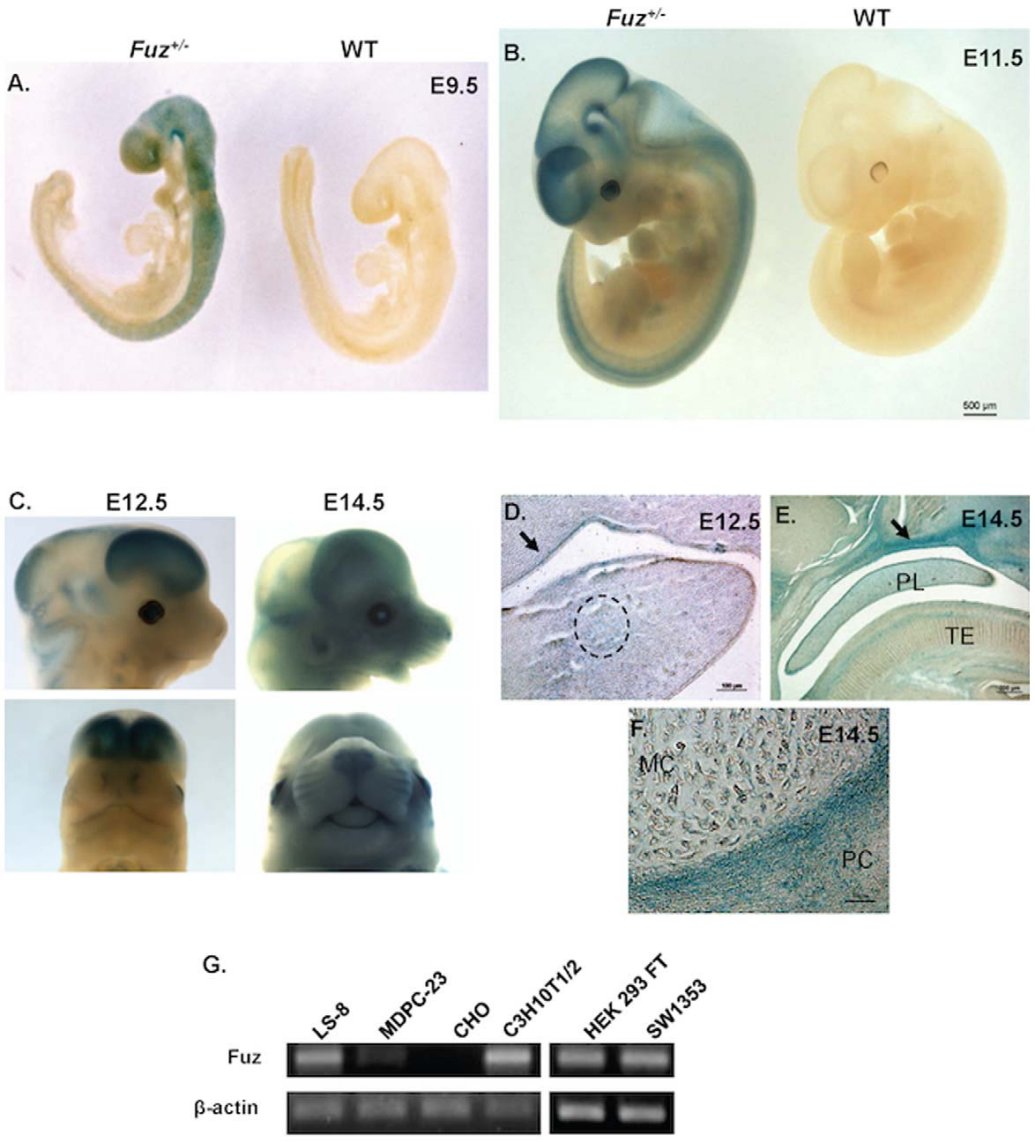
Expression of the *Fuz^LacZ^* allele during mouse craniofacial development. *Fuz^LacZ^* allele expression is shown by X-Gal staining of heterozygous *Fuz^LacZ/+^* embryos. **A**) *Fuz^LacZ^* expression is predominantly in the dorsal tissue at E9.5. **B**) At E11.5, *Fuz^LacZ^* expression is concentrated in the brain, spinal cord and eyes. **C**) At E12.5, *Fuz^LacZ^* expression begins to expand from dorsal to ventral region. Fuz expression in the oral epithelium (arrow), mesenchyme and Meckel's Cartilage is shown in a sagittal section (dotted circle) (**D**). At E14.5 embryos, the ventral expression of *Fuz^LacZ^* is increased while the dorsal expression is relatively decreased (**C**). *Fuz^LacZ^* expression in the oral epithelium (arrow, **E**), Meckel's Cartilage (MC) and the perichondrium (PC) is shown in a sagittal section (**E, F**). **G**) RT-PCR reveals that *Fuz* is highly expressed in LS-8 (oral epithelium), C3H10T1/2 (embryonic fibroblast), HEK 293 FT (embryonic kidney fibroblast) and SW1353 (chondrocyte) cell lines. It has relatively weak expression in MDPC-23 cells (dental mesenchyme), and no expression in CHO cells (ovary). PL, palate; TE, tongue.

X-gal staining of E9.5 *Fuz^LacZ/+^* mouse embryos revealed *Fuz^LacZ^* expression was restricted to the dorsal tissue and brain (Fig. 1A). At E11.5, *Fuz^LacZ^* expression was concentrated in the brain, spinal cord and eyes. However, relatively weak expression was also detected in the heart, limbs and craniofacial region (Fig. 1B). At E12.5, *Fuz^LacZ^* expression in the craniofacial region began to expand from dorsal to the ventral regions (Fig. 1C). At E14.5, *Fuz^LacZ^* expression became stronger and widespread throughout the craniofacial region (Fig. 1C). *Fuz* expression at E12.5 in the oral epithelium, mesenchyme and Meckel's Cartilage is shown in sagittal sections (Fig. 1D). *Fuz^LacZ^* expression at E14.5 in the oral epithelium, mesenchyme, palate (PL), and tongue (TE) has increased from E12.5 (Fig. 1E). Fuz expression in Meckel's cartilage (MC) and the perichondrium (PC) are shown in high magnification sections (Fig. 1F). In addition, we assessed *Fuz* expression in different cell lines by RT-PCR. *Fuz* is highly expressed in LS-8 cells (mouse oral epithelium), C3H10T1/2 cells (mouse embryonic fibroblast), HEK 293 FT cells (human embryonic kidney fibroblast) and SW1353 cells (human chondrocyte). It had relatively weak expression in MDPC-23 cells (mouse dental mesenchyme), and no expression in CHO cells (hamster ovary) (Fig. 1G).

### Severe craniofacial defects in the *Fuz^−/−^* mice

**Figure 2:**
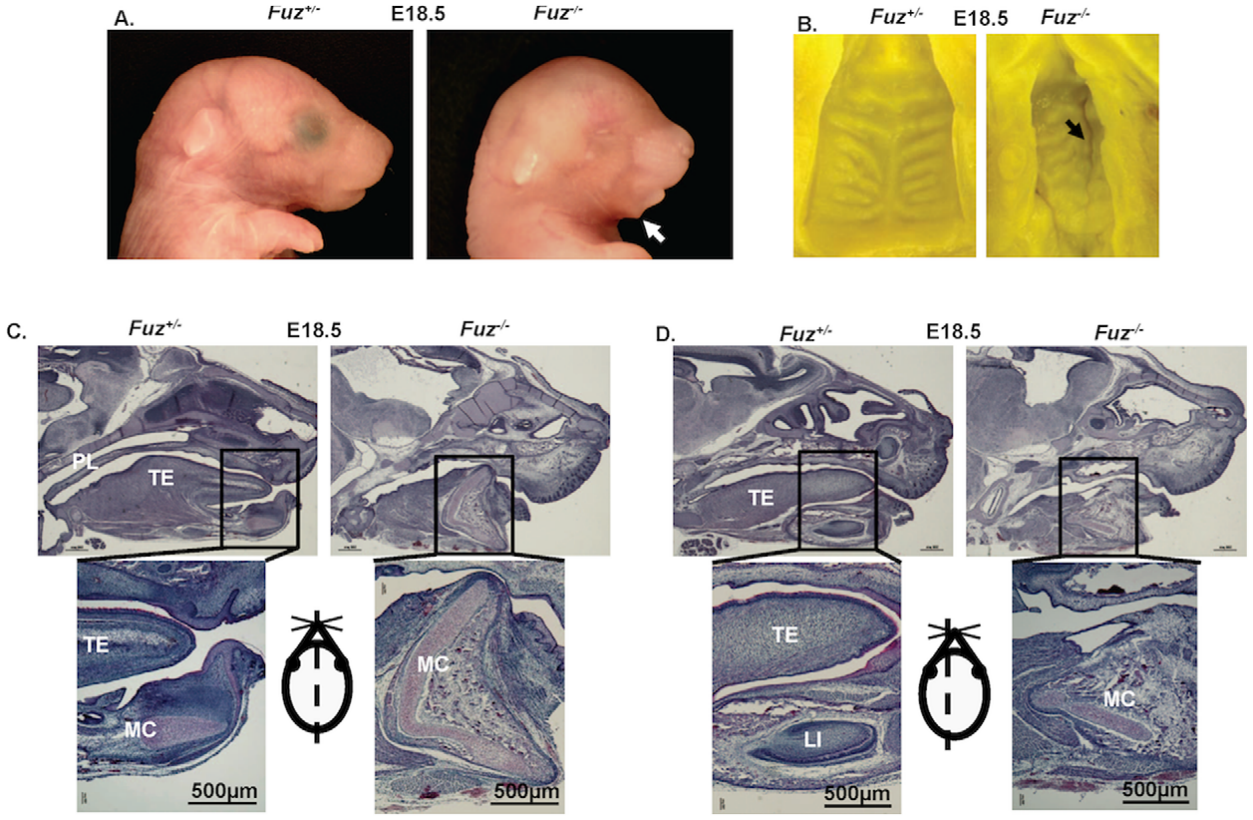
Craniofacial defects associated with a deletion of *Fuz* at E18.5. **A**) E18.5 embryos of heterozygous (*Fuz^+/−^*) and homozygous (*Fuz^−/−^*) mice. The null embryo (*Fuz^−/−^*) has a hypoplastic maxilla and mandible, missing eyes and displaced ears. The white arrow denotes an abnormal bulge in the null mandible. **B**) The cleft palate is shown by the black arrow in E18.5 null embryos (*Fuz^−/−^*). (**C, D**) H&E staining of heterozygous (*Fuz^+/−^*, left) and homozygous (*Fuz^−/−^*, right) head sagittal sections. The bottom panels are higher magnification of the top panels. **C**) At E18.5, the *Fuz* homozygous mandibles are shorter than heterozygotes. Meckel's cartilage expends in the dorsal-ventral axis in addition to anterior-posterior axis and the ventral end of the mutant Meckel's cartilage points down and forms the mandibular bulge. The plane of section is depicted by the dotted line through the mouse drawing. TE, tongue; MC, Meckel's cartilage; LI, lower incisor.

To study *Fuz* function during craniofacial development, we used the *Fuz* null mouse created with a gene trap cassette inserted in the second exon of the *Fuz* gene [7]. The lack of both copies of the *Fuz* gene in homozygotes (*Fuz^−/−^*) was lethal for mice immediately after birth. At E18.5, all null embryos have a hypoplastic mandible and maxilla, and anophthalmia (Fig. 2A). An abnormal bulge in the mandible and a secondary cleft palate are observed in mutant embryos (Fig. 2A,B). Histological analysis on E18.5 embryos revealed further craniofacial defects including a malformed tongue and missing incisors. The ventral bulge of the mutant mandible is due to a hyperplastic and malformed Meckel's cartilage (Fig. 2C,D). The *Fuz^−/−^* mice do not form incisor tooth buds and lower and upper incisor tooth initiation did not begin and the tongue muscles appear to be fused with the mandible. The hyperplastic and malformed Meckel's cartilage suggested an increased proliferation of Meckel's cartilage cells.

**Figure 3:**
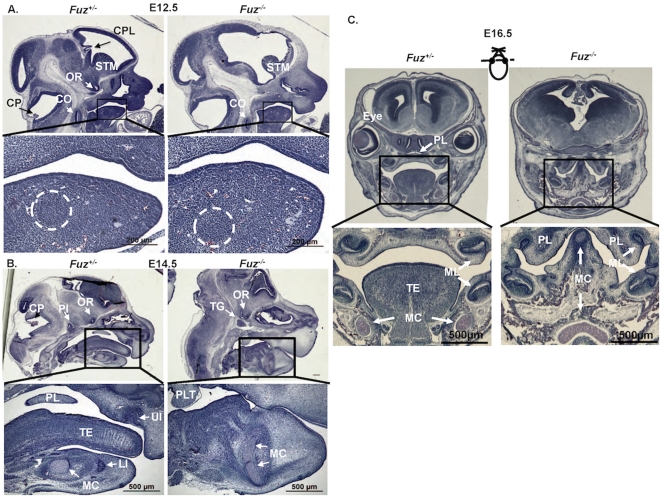
Early stages of craniofacial defects in the *Fuz^−/−^* mice. **A**) E12.5 embryos of heterozygous (*Fuz^+/−^*) and homozygous (*Fuz^−/−^*) mice. The overall structure of the ventral craniofacial region is not severely affected at this stage in the *Fuz* null embryos, due to the low expression of Fuz. However, the dorsal structures including the choroid plexus (CP) differentiating from the roof of fourth ventricle, the choroid plexus extending into the lateral ventricle (CPL), and the optic recess of the diencephalon (OR) are missing in the *Fuz* null mice. The corpus striatum mediale (STM), and the cochlea (CO) are displaced in the *Fuz* null mice. Higher magnification of the mandible is shown in the bottom panels and reveals normal Meckel's cartilage formation at this stage. **B**) At E14.5 the craniofacial defects of the *Fuz* null mice are severe. The tongue muscles are fused with the mandible, the pituitary (PI) is missing and the trigeminal nerve (TG) is seen in its place and the OR is displaced, corresponding to anophthalmia in the mutant. The higher magnification sections showed a lack of upper (UI) and lower incisors (LI), no tongue (TE) and expanded Meckel's cartilage (MC) in the dorsal-ventral axis instead of anterior-posterior axis. The palate is now a piece of displaced palatal tissue (PLT). **C**) E16.5 coronal sections reveal a cleft palate in the *Fuz* null mice. The palate tissue is displaced to the lateral portions of the oral cavity and the secondary palate is not fused, due to the dorsal-ventral and medial-lateral expansion of Meckel's cartilage. The molars (ML) are shown and appear to be grossly normal in the mutant mice. The plane of section is depicted by the dotted line through the mouse drawing.

To examine the developmental progress of craniofacial formation, embryos were harvested at different developmental time points and analyzed. At E12.5, the mandible of the *Fuz^−/−^* embryos appeared normal compared with those of their heterozygous littermates. Meckel's cartilage of the *Fuz^−/−^* embryos had a normal shape and similar size with heterozygous embryos (Fig. 3A). The normal mouse craniofacial structures such as the choroid plexus (CP) differentiating from the roof of fourth ventricle, the choroid plexus extending into the lateral ventricle (CPL), the corpus striatum mediale (STM), the optic recess of the diencephalon (OR) and the cochlea (CO) are present in the E12.5 *Fuz^+/−^* heterozygous mouse (Fig. 3A). These structures are lost in the *Fuz* null mice at this stage, only the cochlea and corpus striatum are observed and these structures are abnormal (Fig. 3A). In contrast, at E14.5 the *Fuz^−/−^* entire mandible is thickened in the dorsal-ventral axis and shortened in the anterior-posterior axis compared with those of heterozygous littermates (Fig. 3B). The trigeminal nerve (TG) appears to replace the pituitary primordium or Rathke's pouch (RP) and many of the brain structures are not developed (Fig. 3B). Meckel's cartilage has begun to elongate in the dorsal-ventral direction instead of the anterior-posterior direction (Fig. 3B). The upper and lower incisor buds have not developed while the tongue had a rudimentary root structure and failed to elongate in the anterior-posterior axis (Fig. 3B). The malformed Meckel's Cartilage not only elongated dorsoventrally but also the dorsal end expanded in the median-lateral axis. An abnormal growth of the palate tissue (PLT) or palate shelf was observed in the E14.5 *Fuz* null mice compared to the normal palate (PL) seen in the heterozygous mice (Fig. 3B). Coronal sections of E16.5 *Fuz^−/−^* mice revealed descending palate shelves (PL) suggesting a delay of elevation, a lack of eyes, however the upper and lower molar (ML) tooth buds have formed (Fig. 3C). Meckel's cartilage has developed in the midline and extends dorsally (Fig. 3C). There were many other defects observed in the nasal cavity, facial bones, cartilage and brain that will not be examined in this report. However, the loss of *Fuz* affects patterning of much of the craniofacial region with a loss of some structures (incisors, tongue, eyes and bone) and an expansion of other structures (Meckel's cartilage).

### Defective craniofacial bone development in *Fuz* null mice

**Figure 4:**
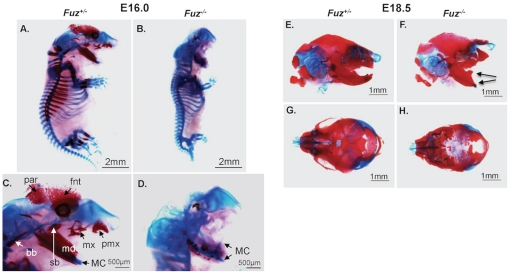
Skeletal defects of *Fuz* null mutant embryos. **A, B**) Upper panels are Alcian Blue/Alizarin Red staining of cartilage and bone skeletal preparations of E16.0 *Fuz* heterozygous embryos (A) and *Fuz* null littermates (B). Ossification is reduced in the *Fuz* null embryos. Lower panels are higher magnification of the head region. The anterior region of Meckel's cartilage (MC) is deformed with an ascending branch. **E–H**) E18.5 head preparations revealing delayed bone formation and defective craniofacial structures in the *Fuz* mutant embryos (arrows denote the malformed and ossified Meckel's cartilage). Frontal (fnt), parietal (par), premaxilla (pmx), maxilla (mx), sphenoid (sb) and basioccipital (bb) bones and other facial bones are missing. Background red staining was due to soft tissues, which were left intact.

To determine if bone formation was affected in the *Fuz* null mice, E16.0 embryos were stained with Alcian Blue/Alizarin Red and compared to their heterozygous littermates (Fig. 4A–D). The blue cartilage stain revealed defective Meckel's cartilage in the *Fuz^−/−^* mice and malformed cartilage of the craniofacial region (Fig. 4B,D). The anterior Meckel's cartilage is deformed with an ascending branch and reduced ossification (red stain) around Meckel's cartilage. Specifically, membranous ossification of the premaxilla (pmx), maxilla (mx), mandible (md), frontal (fnt) and parietal (par) bones are missing at this stage. Ossification of the sphenoid (sb) and basioccipital (bb) bones are also defective in the *Fuz* null mice (Fig. 4D). To determine if craniofacial bone development was delayed or reduced in the *Fuz* null mice we analyzed E18.5 *Fuz* null mice and found a delayed ossification of the craniofacial region compared to heterozygous littermates (Fig. 4E–H). Further research is ongoing to understand the delayed bone development and in this report we focused on the regulatory mechanisms directing Meckel's cartilage formation.

### Increased proliferation of Meckel's cartilage cells in *Fuz* null embryos

**Figure 5:**
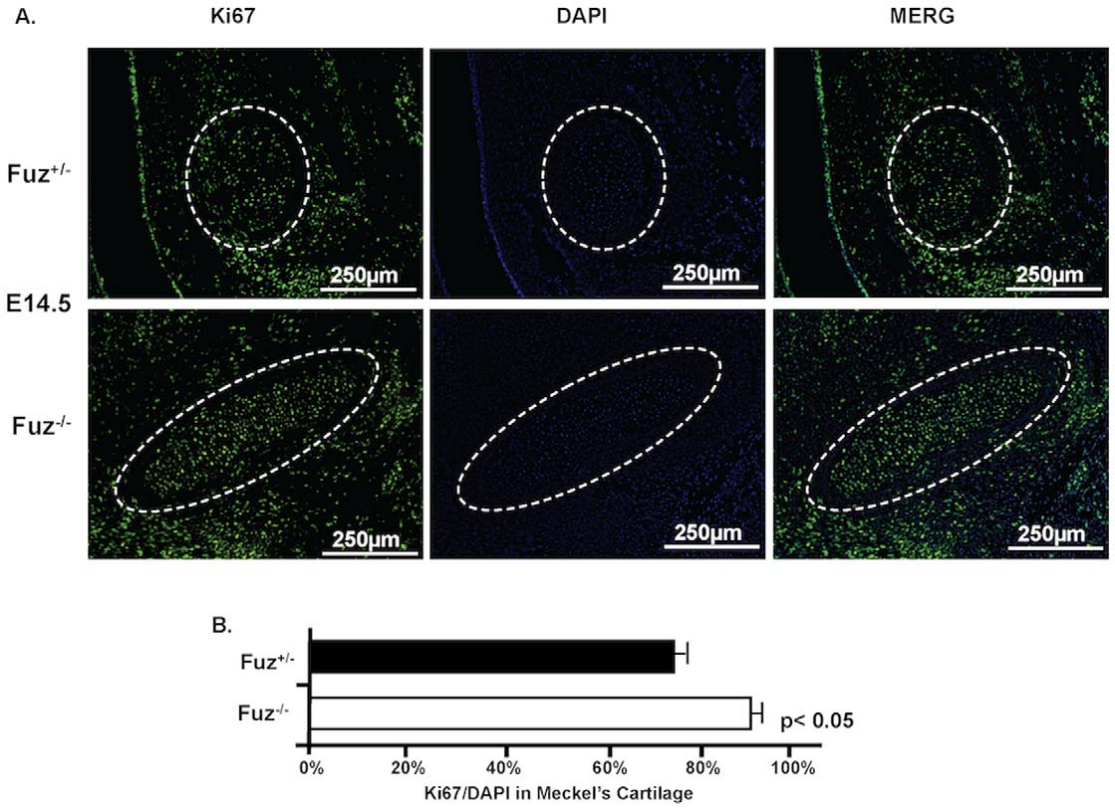
Enhanced cell proliferation in Meckel's cartilage of the *Fuz^−/−^* mandible. **A**) The proliferation of E14.5 Meckel's cartilage was assessed by immunofluorescence with a Ki67 antibody. **B**) The proliferation ratio is calculated by dividing the Ki67 positive cell number with DAPI cell number within Meckel's cartilage. The proliferation of mutant Meckel's cartilage (88%) is significantly increased compared with wild type (75%). Experiments were repeated three times and *p*-value is shown.

Cell proliferation of E14.5 Meckel's cartilage was measured by immunofluorescence with a Ki67 antibody (Fig. 5A). The Ki67 positive cell number to total cell number (DAPI) within Meckel's cartilage was calculated to estimate the proliferation ratio. Compared with a 75% ratio observed in heterozygous Meckel's cartilage, the ratio in null samples was significantly increased to 88% (Fig. 5B). These cells are proliferating at a higher rate compared to *Fuz* heterozygous Meckel's cartilage.

### *Fuz* regulates cilia development in the mandibular mesenchyme

**Figure 6:**
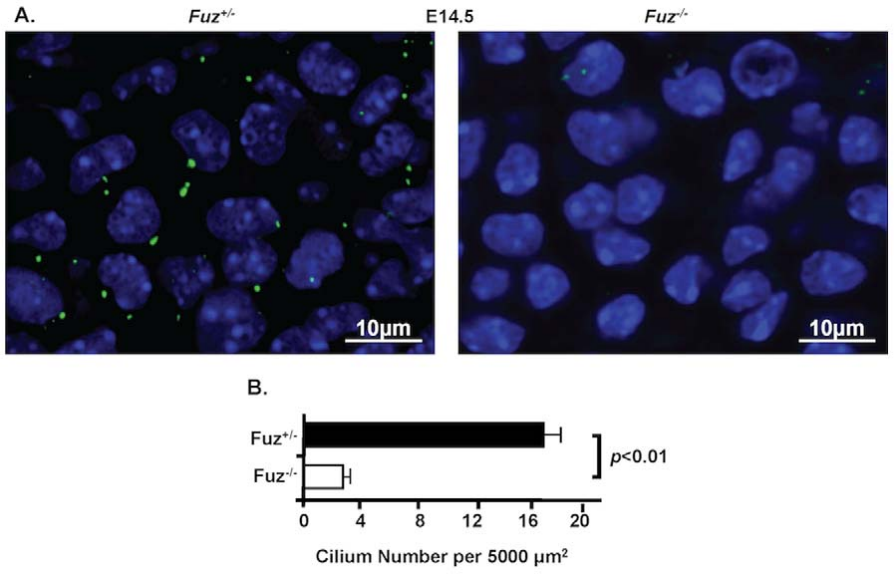
The cilium defect in the *Fuz* null mouse. **A**) The primary cilia are shown by immunofluorescence with an Arl13b antibody in mandible mesenchyme at E14.5. **B**) The amount of cilia in the sagittal sections of *Fuz^−/−^* mandible mesenchyme was quantitated and compared to wild type mandible mesenchyme. Error bars indicate S.E., n = 8, *p*<0.01.

We asked if cilia formation was affected in the mandible by immunofluorescence with an Arl13b antibody in mandible mesenchyme at E14.5. The amount of primary cilium was significantly decreased in the *Fuz^−/−^* mandible mesenchyme (Fig. 6). The average cilium number per 5000 µm^2^ in the sagittal sections of the mutant mandible mesenchyme was 2.9, compared to 16.5 in the wild type. Because Hh signaling is regulated by the primary cilium components, we hypothesized that the Hh signaling was altered due to the cilium defect in the *Fuz* null mouse.

### Down-regulation of Hedgehog signaling in *Fuz^−/−^* embryos

**Figure 7:**
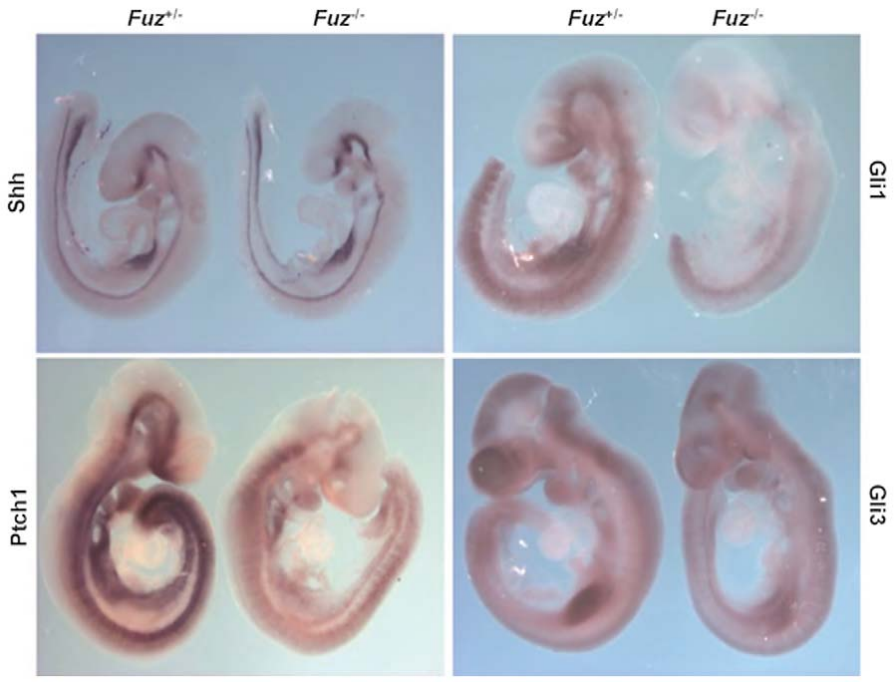
Defective Sonic Hedgehog signaling. Whole-mount *in situ* hybridization assays with indicated probes were performed with E9.5 embryos. The overall decrease of *Ptch1*, and *Gli1* transcript levels are shown.

A loss of primary cilia correlates with an absence of membrane associated Smoothened and failure to activate downstream transcription factors responding to the Hh signal [4], [32], [33]. We asked if sonic hedgehog (Shh) signaling was altered in the craniofacial region of *Fuz* mutant embryos. A whole-mount in situ hybridization assay was performed at E9.5 and showed an overall reduction of *Patched 1* (*Ptch1*), and *Gli1* expression levels while *Shh* levels were unchanged (Fig. 7). Given that *Ptch1* and *Gli1*are indicative of Hh signaling [34], [35], these data revealed a general down-regulation of Hh signaling in the *Fuz* null mouse.

**Figure 8:**
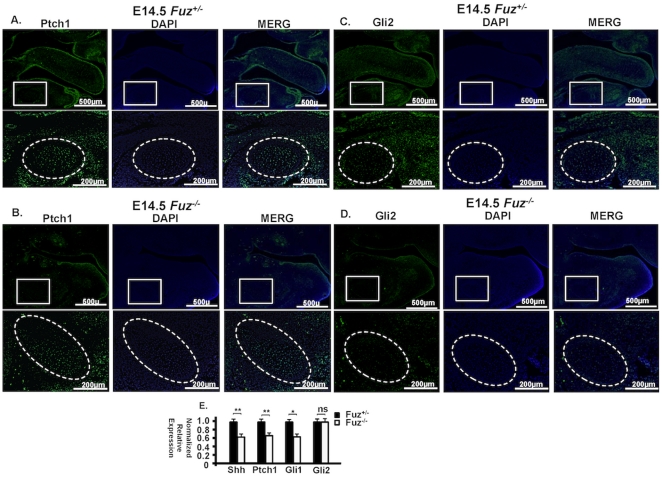
The Hh signaling pathway effectors are decreased in E14.5 *Fuz^−/−^* mouse embryos. **A**) Immunofluorescence of Patched1 in sagittal sections of E14.5 *Fuz^+/−^* heterozygous embryos. Sections in the bottom panels are higher magnification of those in the top panels. Ptch1 is weakly expressed in Meckel's cartilage. **B**) In the *Fuz^−/−^* embryos, Ptch1 expression is decreased overall as well as in Meckel's cartilage. **C**) Immunofluorescence of Gli2 in sagittal sections of E14.5 *Fuz^+/−^* heterozygous embryos. Sections in the bottom panels are higher magnification of those in the top panels. Gli2 is highly expressed in the oral and dental epithelium and mesenchyme, but weakly expressed in the Meckel's cartilage. The expression pattern of Gli2 is similar to Ptch1, but Gli2 has increased expression in the oral mesenchyme compared to Ptch1. **D**) In the *Fuz^−/−^* embryos, Gli2 expression was decreased overall as well as in Meckel's cartilage. **E**) The wild type and *Fuz^−/−^* Meckel's cartilage and surrounding mesenchyme were dissected from E14.5 embryos and the mRNA was extracted, followed by reverse transcription. The real-time PCR was performed with indicated probes. Results are shown as normalized relative expression. βactin served as the reference gene. Experiments were repeated three to five times each from multiple samples. Error bars indicate S.E. *: *p*-values<0.05; **: *p*-values<0.01.

Immunohistochemistry experiments revealed that Ptch1 was highly expressed in the tongue, oral and dental epithelium and mesenchyme, but weakly expressed in Meckel's cartilage in E14.5 heterozygous embryos (Fig. 8A). In the *Fuz* null embryos, Ptch1 expression was decreased overall in the oral cavity as well as in Meckel's cartilage (Fig. 8B). Real-time PCR with mRNA of dissected Meckel's cartilage and surrounding mesenchyme confirmed the down-regulation of Ptch1 (Fig. 8E). Real-time PCR also revealed a significant decrease in *Gli1* transcripts in the *Fuz* null embryos (Fig. 8E). At E14.5, Gli2 had a similar expression pattern with Ptch1 and Gli2 protein was decreased in the null embryos compared to heterozygotes (Fig. 8C,D), though its transcript level was not significantly changed (Fig. 8E). *Shh* transcript levels were also reduced in the null embryos suggesting that *Fuz* was required for the maintenance of Hh signaling. Hh signaling is essential for establishing and maintaining dorsal-ventral patterning and required for incisor development. The disrupted Hh signaling provides a possible explanation for the malformed Meckel's cartilage and the missing incisors. Hh signaling is also able to stimulate cell proliferation by activating *Cyclin D1* (*Ccnd1*) and *Cyclin D2* (*Ccnd2*) expression [36]. However, the decreased Hh signaling is contrary to the increased proliferation of Meckel's cartilage. We hypothesized that another mechanism must be involved in the enhanced proliferation of Meckel's cartilage.

### Increased Wnt/β-catenin signaling in *Fuz^−/−^* embryos

**Figure 9:**
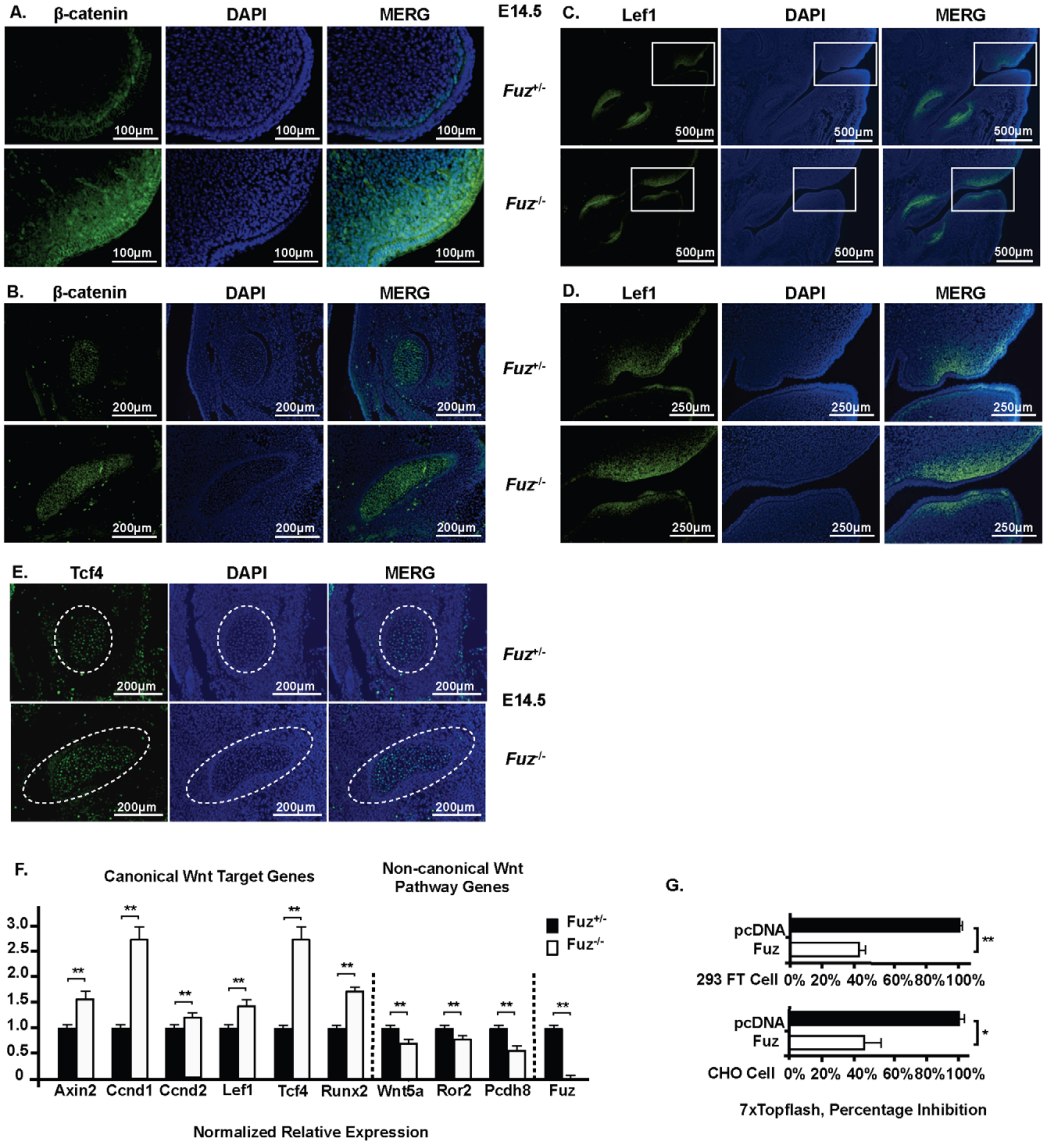
Wnt/β-catenin signaling is increased in the *Fuz* null mice. Immunofluorescence on sagittal sections of E14.5 embryos. **A**) β-catenin expression was increased in the oral epithelium and mesenchyme in mutant (*Fuz* ^−/−^) embryos. The bottom panels are the *Fuz^−/−^* embryos. **B**) β-catenin expression was also increased in the *Fuz* null Meckel's cartilage (bottom panel), compared to the heterozygote (*Fuz* ^+/−^, top panel) samples. **C**) Lef-1 expression in the dental epithelium, oral and dental mesenchyme at E14.5. Lef-1 expression increased in the *Fuz* null oral mesenchyme (bottom panel). **D**) These are higher magnification of the boxed areas in C. Lef-1 expression was expanded in the *Fuz^−/−^* mice oral mesenchyme. **E**) The expression of *Tcf4* (*Tcf7l2*) was increased in *Fuz* mutant Meckel's cartilage (bottom panels) at E14.5 compared with wild type samples (top panels). **F**) Real-time PCR with mRNA from dissected E14.5 Meckel's cartilage and surrounding mesenchyme. Canonical Wnt target gene expression was increased whereas non-canonical Wnt pathway gene expression was decreased. β-actin served as the reference gene. Experiments were repeated three to five times each from multiple samples. **G**) Topflash reporter activity was repressed by co-transfection of *Fuz* in HEK 293FT and CHO cells. The activities are shown as mean fold activation compared to reporter activation co-transfected with pcDNA3.1 empty vector and normalized to SV-40 β-galactosidase activity. Error bars indicate S.E. *: *p*-values<0.05; **: *p*-values<0.01.

Wnt/β-catenin signaling is regulated by the basal component of the primary cilium [29], [30] and Hh signaling interacts with Wnt/β-catenin signaling during multiple developmental programs [37], [38], [39]. We asked if Wnt/β-catenin signaling was altered in the *Fuz* null embryos, which could affect cell proliferation. Immunofluorescence with a β-catenin antibody was performed using sagittal sections of E14.5 embryos. These experiments revealed that β-catenin was increased in the *Fuz^−/−^* oral epithelium, mesenchyme (Fig. 9A) and Meckel's cartilage (Fig. 9B), compared to those of heterozygous littermates. Because Lef-1 is a transcription factor whose activity and expression are positively regulated by Wnt/β -catenin signaling, we asked if Lef-1 expression was modulated in the *Fuz* null mutants. Lef-1 is normally expressed in the oral and dental epithelium and mesenchyme at E14.5 (Fig. 9C,D, upper panels). In the null embryos Lef-1 expression in the oral mesenchyme was increased (Fig. 9C,D, lower panels), confirmed by Real-time PCR (Fig. 9F). *Tcf4* (*Tcf7l2*) expression is also regulated by Wnt/β-catenin signaling [40], and immunofluorescence assays revealed increased Tcf4 in *Fuz* null Meckel's cartilage at E14.5, and further confirmed by Real-time PCR (Fig. 9E,F). Inspection of a group of Wnt/β -catenin target genes revealed increased expression of *Axin2*, *Cyclin D1*, *Cyclin D2* and *Runx2* in *Fuz^−/−^* Meckel's cartilage (Fig. 9F), [41], [42], [43], [44]. On the contrary, non-canonical Wnt signaling including *Wnt5a*, *Ror2*, and their downstream target *Pcdh8* (known as *Papc* in Xenopus) [45] were down regulated in *Fuz^−/−^* Meckel's cartilage (Fig. 9F). As a control real-time PCR demonstrated a lack of *Fuz* transcripts in the null embryos (Fig. 9F). These data indicate that *Fuz* acts as a repressor of Wnt/β-catenin signaling during craniofacial development. The increased *Cyclin D1* and *D2* provide a possible explanation of the enhanced cell proliferation in the *Fuz^−/−^* Meckel's cartilage.

To confirm the repressor role of *Fuz* on Wnt/β-catenin signaling, *Fuz* expression plasmid was co-expressed with the 7xTopflash reporter in both HEK 293 FT cells and CHO cells. Co-transfection of Fuz with the 7xTopflash reporter resulted in a 50% decrease in Topflash reporter activity, compared to that with the control vector in both cell lines (Fig. 9G). The result was consistent between 293 cells, which has high endogenous *Fuz* expression and CHO cells which do not endogenously express *Fuz*. These results indicate a repressor role of Fuz on Wnt/β-catenin signaling.

### β-catenin directly activated the *Fuz* promoter

**Figure 10:**
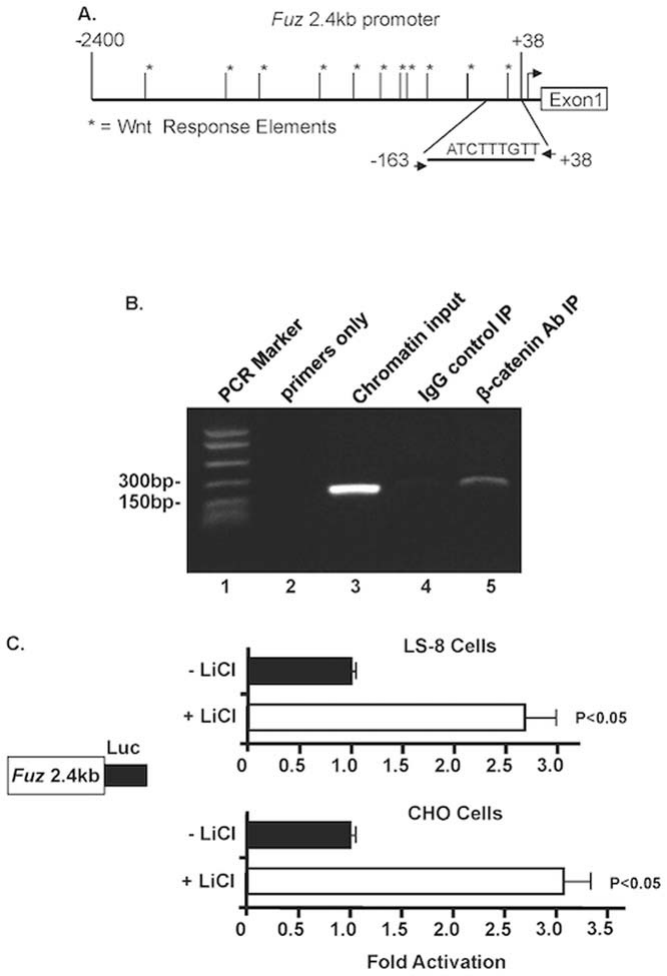
β-catenin activates the *Fuz* promoter. **A**) A schematic of the *Fuz* 2.4 kb promoter with eleven Wnt response elements (*Lef/Tcf* binding sites). Chromatin immunoprecipitation assay reveals endogenous β-catenin associated with the *Fuz* promoter chromatin in LS-8 cells. The location of the PCR primers are shown in A. **B**) A gel with specific PCR products from the immunoprecipitated chromatin and controls are shown. **C**) The *Fuz* 2.4 kb promoter was transfected into LS-8 and CHO cells and LiCl (10 mM) was added to the cell culture medium. The activities are shown as mean fold activation compared to reporter activation without LiCl and normalized to SV-40 β-galactosidase activity. Error bars indicate S.E.

Sequence analyses revealed eleven Wnt response elements (*Lef/Tcf* binding sites) within the murine *Fuz* 2.4 kb promoter (Fig. 10A). A chromatin immunoprecipitation (ChIP) assay demonstrates endogenous β-catenin associating with the *Fuz* promoter. Non-transfected LS-8 cells were used as these cells endogenously express Lef-1, β-catenin and Fuz. The *Fuz* promoter chromatin was amplified by PCR using primers specific for the *Fuz* promoter flanking the *Lef/Tcf* binding site (Fig. 10A,B). The primers amplified a 201 bp product from the chromatin input and antibody IP (Fig. 10B, lanes 3 and 5, respectively). The primers did not produce a PCR product from primers only or normal rabbit IgG IP control (Fig. 10B, lanes 2 and 4, respectively). To test whether this association could lead to functional activity, the *Fuz* 2.4 kb promoter was cloned into a luciferase vector and transfected into LS-8 and CHO cells. Addition of LiCl (10 mM) to the cell culture medium stimulates β-catenin nuclear localization and caused significant increase of *Fuz* promoter activity in both cell lines (Fig. 10C). These results demonstrate that β-catenin directly targets the *Fuz* promoter and activates its transcription. Combined with the Fuz repression of Wnt/β-catenin signaling, we conclude that Fuz constitutes a negative feedback loop that controls Wnt/β-catenin signal activity.

### *Sox9* expression is increased in *Fuz^−/−^* Meckel's cartilage

**Figure 11:**
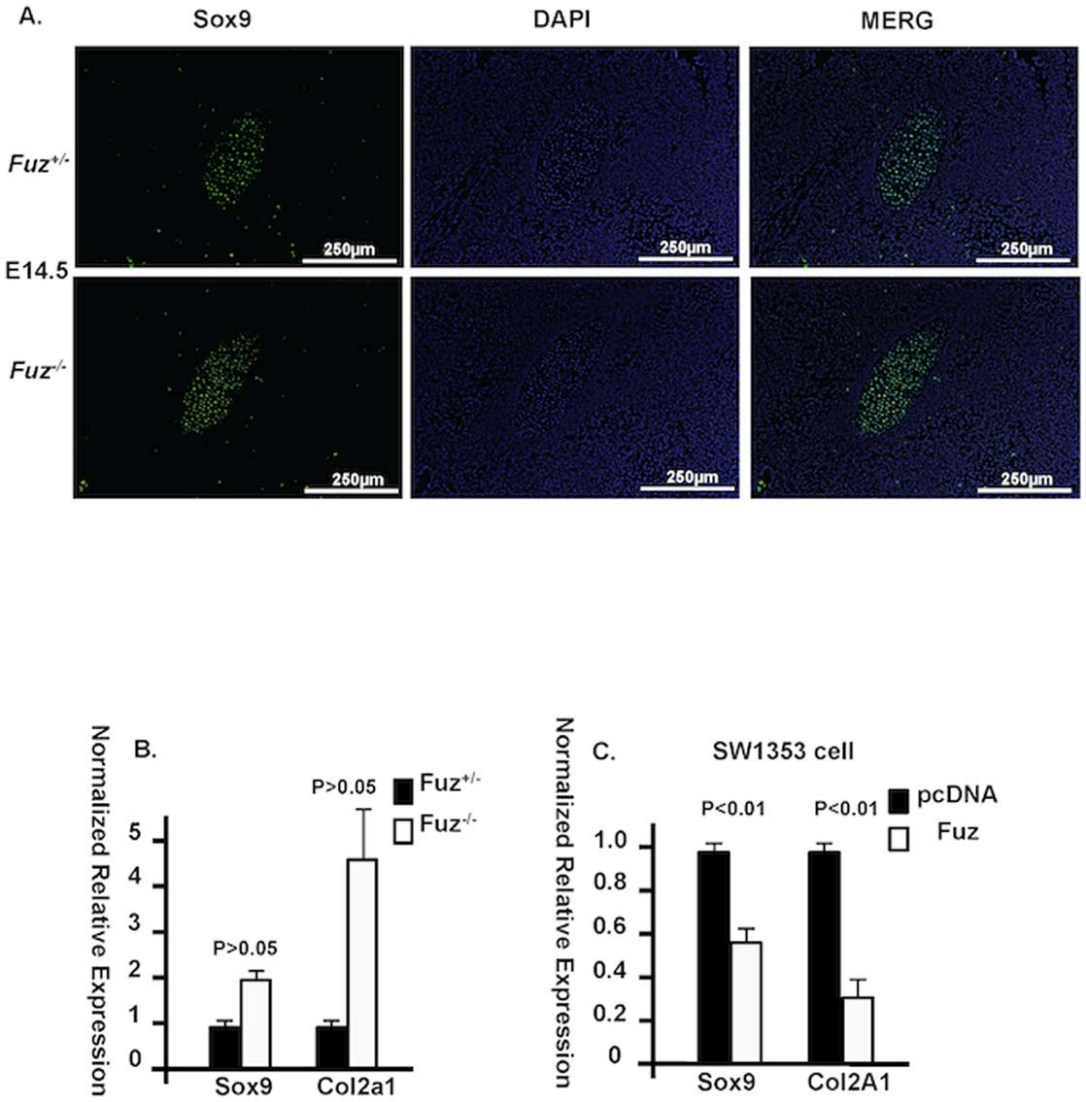
Sox9 expression was increased in *Fuz^−/−^* Meckel's cartilage. **A**) Sox9 expression was expanded in E14.5 *Fuz^−/−^* Meckel's cartilage shown by immunofluorescence (bottom panel) compared to heterozygotes (*Fuz^+/−^*, top panel). **B**) Real-Time PCR revealed increased *Sox9* expression as well as *Type II Collagen* (*Col2a1*) in *Fuz* null Meckel's cartilage at E14.5. **C**) Transfection of *Fuz* results in reduced *SOX9* and *COL2A1* expression in chondrocyte (SW1353) cells compared to those transfected with control vectors. Error bars indicate S.E.M.

A previous study reported that Wnt/β-catenin signaling repressed *Sox9* expression both in vitro and in vivo [46]. Given the increased Wnt/β-catenin signaling in the *Fuz* null mice, we expected reduced expression of *Sox9* in *Fuz* null Meckel's cartilage. However, immunofluorescence with Sox9 antibody in E14.5 Meckel's cartilage revealed that *Sox9* expression was increased in the null embryos (Fig. 11A). This increase was validated by Real-time PCR using RNA from E14.5 Meckel's cartilage (Fig. 11B). Type II Collagen (*Col2a1*) is a downstream target gene of Sox9 [47]. *Col2a1* expression was also increased in *Fuz* null Meckel's cartilage measured by Real-time PCR (Fig. 11B). Over-expression of Fuz in SW1353 cells confirmed the repression of *Sox9* expression. The human chondrocyte cell line SW1353 was transfected with the Fuz expression vector and total RNA was harvested two days after transfection. Real-time PCR revealed that endogenous *SOX9* and *COL2A1* expression was significantly repressed in the Fuz transfected cells compared to those with control vector. These results indicate that Fuz represses *Sox9* and cartilage expansion during craniofacial development. Loss of *Fuz* leads to increased *Sox9* expression in the null mice, which maintains cartilage expansion in the presence of increased Wnt/β-catenin signaling.

## Discussion

In this study we have analyzed the role of the *Fuz* gene during craniofacial development. *Fuz* loss of function analyses revealed a critical function of this gene in the development of multiple craniofacial tissues and structures. *Fuz* is required for the formation of eyes, bone, tongue and incisors. These affected organs and craniofacial structures show essential roles of *Fuz* in tissue patterning and cell proliferation.

### *Fuz* regulates Hh signaling

In the *Fuz* null mice, down regulation of Hh signaling has been shown in the neural tube and limb buds [7], [8]. In this study we have shown a decrease in Hh downstream gene expression in early and late craniofacial development. Hh signaling is essential for dorsal-ventral patterning and incisor development, but apparently not for molar development, as the molar tooth buds form normally in the *Fuz^−/−^* mice. A loss of Hh signaling in the oral epithelium results in a lack of epithelial cell proliferation and tooth bud formation [48]. This would explain the absence of incisor development, but not molar development. We suspect that this is a timing issue as incisors develop earlier than molars, however more experiments are required to understand the defect. Hh signaling is required for mouse brain and craniofacial morphogenesis and loss or gain of Hh function is associated with midline facial anomalies [49], [50]. However, the overall craniofacial defects observed in the *Fuz* null mice do not resemble the other cilia related gene defects or *Shh* mutant mice, with the exception of brain and cleft palate defects. A recent report revealed a role for *Fuz* in the development of hair follicles [51]. Hair follicle development was impaired due to inhibition of cilia formation and Hh signaling, revealing a direct role for *Fuz* in regulating Hh signaling through defective cilia formation. Interestingly, Fuz regulated Hh signaling does not appear to regulate Meckel's cartilage growth but may affect patterning and placement of Meckel's cartilage in the mandible. Thus, *Fuz* could be regulating Hh signaling which in turn restricts the normal growth and patterning of Meckel's cartilage by influencing the surrounding tissue formation (such as incisor development) and other craniofacial structures.

### *Fuz* regulates Wnt signaling

In the *Fuz* null mice, the Wnt/β-catenin pathway was up regulated whereas the non-canonical Wnt5a/Ror2 pathway was down regulated. These data suggest that *Fuz* is a potent repressor of Wnt/β-catenin signaling. The data also indicate that *Fuz* is critical to maintain the non-canonical Wnt signaling pathway, which is overlapped with PCP signaling. It provides a possible explanation of the neural tube defect (a typical phenotype in non-canonical Wnt deficient mice) previously reported in *Fuz* null mice [7], [52]. The increased Meckel's cartilage growth in the *Fuz^−/−^* mice can be attributed to increased Wnt/β-catenin signaling. Thus, PCP, Wnt/β-catenin and Hh signaling pathways may converge to provide cues for and instruct specific cell differentiation, tissue patterning and morphogenesis.

*Fuz* regulation of Wnt/β-catenin signaling may be mediated by primary cilia and interaction of multiple signaling pathways. Previous studies have reported that the basal component of primary cilium could repress Wnt/β-catenin signaling through interaction of Dishevelled protein [29], [30]. The loss of *Fuz* could cause the secondary up-regulation of Wnt/β-catenin signaling. Previous studies have shown that Hh signaling antagonizes Wnt/β-catenin during development of the tongue and cartilage [37], [38], [53]. The increase in Wnt/β-catenin signaling could be secondary to decreased Hh signaling in the *Fuz* null mice. Non-canonical Wnt signaling is known to antagonize the canonical Wnt/β-catenin activity [17], [45]. In particular, *Wnt5a* was shown to repress Wnt/β-catenin signaling in the limb buds [54]. An in vitro study revealed that the *Ror2* receptor was necessary for *Wnt5a* repression of Wnt/β-catenin activity [55]. In the *Fuz* null mice, down-regulation of *Wnt5a* and *Ror2* could attribute to activation of Wnt/β-catenin signaling as well. Whether it is one of those mechanisms or a combination requires further investigation.

### Primary cilia defects associated with increased Hh signaling

In contrast to loss of *Fuz*, mice mutants for cilia intraflagellar transport (IFT) proteins; IFT88/polaris and Kif3a have increased Hh activity [50], [56]. Mice with mutations in IFT88 lack cilia on all cells and present with severe neural tube defects, polydactyly, asymmetry defects and ectopic tooth formation [56], [57], [58]. *Kif3a* mice mutants display a range of similar developmental defects and a conditional knockout of *Kif3a* in the neural crest results in an increase in Hh activity associated with truncated cilia [50]. However, other groups have reported that mutations in IFT proteins including Kif3a demonstrate decreased Hh signaling [59], [60], [61]. These differences could be attributed to the differential tissue expression of the Gli proteins during development [50], [56], [60], [62], [63]. Our data reveal *Fuz* expression is predominantly expressed in the oral and dental epithelium at early stages and in epithelial cell lines. The *Fuz^−/−^* mice do not present with a wide facial prominence, which is indicative of increased Hh signaling [50]. Furthermore, as a PCP effector it may play an extended role in regulating signaling pathways and gene expression independent of cilia formation. However, unlike the Wnt and Hh signaling mechanisms, which act at the level of transcription, the PCP pathway controls cell morphology [17], [45]. It is not inconceivable that changes in cell morphology induce changes in signaling mechanisms. The *Fuz^−/−^ mice* have reduced and truncated cilia, but not a complete loss of primary cilia. The presence of truncated cilia in the *Fuz* null mice may play a limited role in the signaling activities. Fuz interacts with a Rab-similar GTPase (RSG1) to affect trafficking from the cytoplasm to basal bodies and to cilia tips [7]. Fuz may have a bigger role in exocytosis than ciliogenesis and could promote a cell type-specific regulation of signaling mechanisms and gene expression [7], [17], [64]. Transport and exocytosis facilitated by Fuz maybe be more important and explain the mouse craniofacial phenotypes of the *Fuz* null mice more than defective cilia.

#### Craniofacial Bone and Meckel's Cartilage Defects

Runx2 plays a major role in bone development and specifically intramembranous bone formation during craniofacial development. *Runx2* null mice have a complete loss of bone tissue and regulation of *Runx2* dosage during development can result in the cleidocranial dysplasia phenotype [65]. These hypomorphic *Runx2* mutant mice have reduced ossification of the calvarial bones, reduced basisphenoid bone and non-osseous tissue between the parietal bones, similar to the *Fuz* null mice [65]. However, *Runx2* expression is increased in the *Fuz* null mice and *Runx2* over-expression newborn mice do not reveal defects in calvarial bone development [66]. It is not known if defects occurred at earlier stages of development in these mice. The *Fuz* null mice demonstrated a delayed calvarial bone development, which may appear normal at later stages of newborn mice (*Fuz* null mice die at birth, we are unable to determine if bone development is normal at later stages). We speculate that increased *Runx2* expression may compensate for reduced *Hh* expression, which is also required for bone development.

Meckel's cartilage is a transient structure responsible for mandible formation and undergoes endochondral-like ossification to produce several bones during craniofacial development [67], [68], [69], [70], [71]. Meckel's cartilage is derived from ectomesenchymal cells from cranial neural crest (CNC) cells and non-CNC-derived cells [72], [73], [74]. Sox9 is a critical transcription factor required for cartilage formation and mutations in *Sox9* are associated with campomelic dysplasia, which is characterized by skeletal defects and cranial dismorphology [75], [76], [77]. Sox9 is expressed early in during development in the cranial neural crest cells and regulates Col2a1 required for chondrocyte differentiation and Sox9 appears to be a master regulator of cartilage development [78], [79]. *Sox9* null mice die midway through gestation, however the *Sox9* conditional knockout with *Wnt1-Cre* reveal craniofacial defects including a short mandible and a large cleft palate [80], [81]. Sox9 is required for Meckel's cartilage growth and inactivation of *Sox9* leads to reduced Meckel's cartilage growth. *Sox9* and *Col2a1* expression are increased in the *Fuz* null mice, which correlates with increased Meckel's cartilage growth. The expanded Meckel's cartilage would cause increased endochondral ossification as well giving rise to the boney mandible structure observed in the *Fuz* null mouse mandible. *Sox9* expression also occurs in the mouse head skeleton and some of the bone defects could be due to the activity of Sox9 in these tissues [77]. TGF–β signaling regulates chondrogenesis during mandible development and may be regulated by Fuz [82], [83]. We are currently exploring these mechanisms.

#### Human Ciliopathies

There are multiple ciliary genes and proteins associated with ciliopathies, (for reviews, [22], [84], [85]). Human *FUZ* mutations have not been reported or associated with syndromes with known ciliary pathology. However, Oral-Facial-Digital syndrome, type1 (OFDI; OFD 1; OMIM311200) appears to represent similar phenotypic features to the craniofacial defects associated with the *Fuz* null mouse [86]. Although the tongue is present but is malformed in these patients. This syndrome is caused by mutations in the *Cxorf5* transcript termed *OFD1* [87]. It belongs to a group of developmental disorders known as oral-facial-digital syndromes and is characterized by malformations of the oral cavity, face and digits [88]. *OFD1* is also mutated in X-linked Joubert syndrome [89]. OFD1 is a centrosomal protein localized at the basal bodies of the primary cilia. The mouse *OFD1* mutants reproduce the main features of the human syndromes and have some features not observed in the *Fuz* null mouse [90], [91], [92]. OFD1 is nuclear localized and part of the human TIP60 chromatin-remodeling complex [93]. Recent data reveals that Fuz is not in the nucleus but distributed throughout the cytoplasm (unpublished data, Amendt laboratory). Fuz does not appear to be associated with the plasma or nuclear membranes or specific organelles. The role of *FUZ* in human disorders and the molecular mechanisms of *FUZ* are currently being investigated.

**Figure 12:**
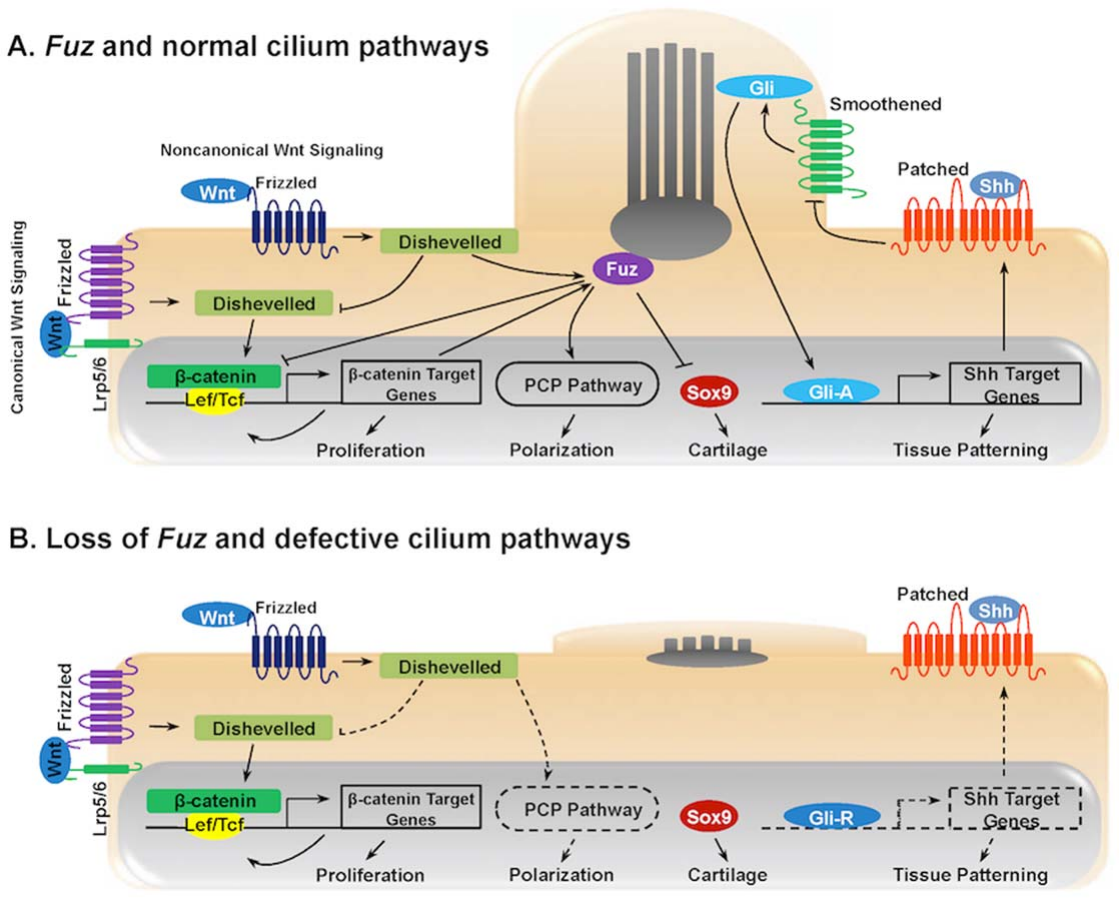
A tentative model linking *Fuz* to Shh, Wnt and PCP signal pathways. **A**) Fuz acts downstream of Frizzled and Dishevelled to regulate PCP signaling. Non-canonical Wnt signaling represses Wnt/β-catenin signaling and Fuz is involved in a negative feedback loop of Wnt/β-catenin signal regulation. Fuz also regulates assembly of the apical actin cytoskeleton, which is critical for ciliogenesis. Cilia formation can be directly linked to Hedgehog signaling and Gli transcription factor activation. Activated Gli factors then regulate Hedgehog target genes. **B**) Loss of *Fuz* leads to impaired PCP signaling and up-regulation of Wnt/β-catenin signaling. In addition, it results in impaired actin cap and cilia, which inhibits the activation of Gli transcription factors and weakens Hedgehog signaling. Loss of *Fuz* also results in the up-regulation of Sox9.

In summary, *Fuz* is critical for Hh, PCP, and Wnt signaling regulation and may serve to connect these distinct signaling pathways. A tentative model linking *Fuz* to Hh, Wnt and PCP signal pathways based on our data and others are shown in figure 12. Fuz acts downstream of Frizzled and Dishevelled to regulate PCP signaling. Non-canonical Wnt signaling represses Wnt/β-catenin signaling and Fuz is involved in a negative feedback loop of Wnt/β-catenin signal regulation. Loss of *Fuz* leads to impaired PCP signaling and up-regulation of Wnt/β-catenin signaling. In addition, it results in impaired actin cap and cilia, which inhibits the activation of Gli transcription factors and weakens Hh signaling. Loss of *Fuz* also causes up regulation of Sox9. These factors together contribute to the complex defects in craniofacial structures, delayed bone development and the hyperplastic and malformed Meckel's cartilage.

## Materials and Methods

### Ethics Statement

All animals were housed at the Institute of Biosciences and Technology under the care of the Program of Animal Resources, and were handled in accordance with the principles and procedure of the Guide for the Care and Use of Laboratory Animals. All experimental procedures were approved by the Texas A&M Health Science Center, Institutional Animal Care and Use Committee. Protocol number 09001, mouse models for tooth development.

### Animals

The *Fuz* gene trap mice were generated by the Texas A&M Institute for Genomic Medicine and were described previously [7]. These mice were maintained in the C57BL/6 background. *Fuz^LacZ^* targeted ES cells corresponding to clone PG00134_Z_E03_2 were purchased from the Knock Out Mouse Project (KOMP) Repository and injected into blastocysts by the Texas A&M Institute for Genomic Medicine. Four chimeras were obtained and mated to wild-type C57BL/6 mice. Two chimeras yielded germ-line transmission. Offspring from both chimeras were used for X-gal staining and exhibited the same expression pattern. Embryos were collected at various time points, considering the day of observation of a vaginal plug to be embryonic day (E) 0.5. Genotyping PCR primers for *Fuz^+/−^* and *Fuz^−/−^* mice and embryos were described previously [7]. Genotyping PCR primers for *Fuz^LacZ^* are as below: forward, 5′- CTCTCCTGGCAGCATGTCCT -3′; reverse, 5′- TCCTCCTACATAGTTGGCAGTG -3′. The resulting PCR product represents the *LacZ* knockin allele, whereas the wild-type allele does not generate any products. All PCR products were sequenced to confirm their identity.

### LacZ staining

Whole embryos of different stages were fixed for 20–40 minutes at room temperature in the fix solution (0.2% glutaraldehyde, 2% formaldehyde, 2 mM MgCl_2_, 5 mM EDTA pH 8.0 and 100 mM NaH_2_PO_4_ pH 7.3) and washed three times in rinse solution (0.2% Nonidet P-40 and 0.1% sodium deoxycholate, 100 mM NaH_2_PO_4_ pH 7.3 and 2 mM MgCl_2_). Embryos were stained for 72 hour at 37°C in staining solution (1.65 mg/ml potassium ferricyanide, 1.84 mg/ml potassium ferrocyanide, 2 mM MgCl_2_, 1 mg/ml X-gal in rinse solution), rinsed in PBS and postfixed in 4% paraformadehyde. Heads of both E12.5 and E14.5 embryos were dehydrated through alcohol, embedded in paraffin and sectioned at 16 µm thickness.

### Histology and immunofluorescence

Samples were fixed in 4% paraformadehyde, dehydrated and embedded in paraffin wax. Sections were cut (7 µm) and stained with Hematoxylin and Eosin. Some embryos were fixed in Bouin's solution and subjected to Wilson section. Immunofluorescence was done on 7 µm paraffin sections with standard procedure. Antigen retrieval was done by boiling samples in 10 mM Sodium Citrate, pH 6.0. Antibodies were obtained and diluted as follows: Ki67 (Abcam, ab15580-100) 1∶500; Ptch1 (Abcam, ab53715) 1∶300; Gli2 (Abcam, ab7195) 1∶300; β-catenin (Upstate, 06-734) 1∶500; Lef1 (Cell Signaling, C12A5) 1∶500; Tcf4 (Cell Signaling, C48H11) 1∶500; Sox9 (Santa Cruz, sc-20095) 1∶500. Secondary antibodies Alexa Fluor 488 goat anti-rabbit HCA were from Invitrogen (A11034) and used at 1∶500 dilution. The Arl13b antibody (Abcam, ab83879) for cilia staining was used at 1∶500 and visualized using a Zeiss Axiovert 200 confocal microscope. Skeletal defects are shown by using Alcian Blue/Alizarin Red staining of cartilage and bone in mouse (Cold Spring Harb Protoc; 2009).

### Real-time PCR analyses

Meckel's cartilage and surrounding mesenchyme were dissected from E14.5 mouse embryos. The Real-time PCR was performed with different probes listed in the table 1. Experiments were repeated three times each from multiple samples and p-values are shown. Total RNA was extracted using the RNeasy mini kit from Qiagen. Total RNA was reverse transcribed into cDNA by iScript Select cDNA Synthesis kit (BioRad). Real-time PCR was carried out in a total reaction of 25 µl containing 12.5 µ iQ SYBR Green Supermix, 0.1 µM forward primer, 0.1 µM reverse primer, 0.25 µl cDNA template in the MyiQ Singlecolor Real-Time Detection System and analyzed by the MyiQ Optical System Software 2.0 (BioRad). The Real-time PCR was performed with gene specific probes. β-actin served as a reference gene. The thermal cycling profile consisted of 95°C for 4 min followed by 40 cycles of denaturation at 95°C for 30 sec, annealing at 60°C for 30 sec and elongation at 72°C for 18 sec. Samples were run in triplicate. Experiments were repeated three times each from multiple samples and *p*-values were calculated. No-template control was run in each experiment. Melting curve analyses were performed to confirm amplification specificity of the PCR products. All PCR products were sequenced to confirm their identity.

**Table 1:** 

Mouse									
Shh	F	5′	AAGCTCACATCCACTGTTCT	3′	Runx2	F	5′	AACTTCCTGTGCTCCGTGCT	3′
	R	5′	GTAAGTCCTTCACCAGCTTG	3′		R	5′	GCCATGACGGTAACCACAGT	3′
Ptch1	F	5′	GTGAGGAGCTCAGGCAATAC	3′	Wnt5a	F	5′	GAGTTCGTGGACGCTAGAGA	3′
	R	5′	GGAGGCTGATGTCTGGAGT	3′		R	5′	GAGCCAGACACTCCATGACA	3′
Gli1	F	5′	GAGAACCTTAGGCTGGATCA	3′	Ror2	F	5′	TTCTTCCTCGTCTGCATGTG	3′
	R	5′	GACTGTGTAAGCAGAGCTCA	3′		R	5′	CCGAGCTCCTCCATGAACCT	3′
Gli2	F	5′	GAAGCTCAAGTCACTGAAGG	3′	Pcdh8	F	5′	TTCAATGACAGTGACTCGGA	3′
	R	5′	ACTTCGGTCAGCTCTGGTAG	3′		R	5′	CTCCAGCAGCGATCAGAATG	3′
Axin2	F	5′	ACAGGAACCACTCGGCTGCT	3′	Fuz	F	5′	CACTTGGAACTGCGACGCTG	3′
	R	5′	AAGTAGGTGACAACCAGCTC	3′		R	5′	CACGAGATAACAGGCTCTGG	3′
Ccnd1	F	5′	CTGCGATGCAAGGCCTGAAC	3′	Sox9	F	5′	TTCCTCCTCCGGCATGAGT	3′
	R	5′	GCGCAGGCTTGACTCCAGAA	3′		R	5′	CCTCTCGCTTCAGATCAACT	3′
Ccnd2	F	5′	GAGCTGCTGGCCAAGATCAC	3′	Col2a1	F	5′	GGCTCCAATGATGTAGAGATG	3′
	R	5′	GACTTGGATCCGGCGTTATG	3′		R	5′	GGAGGTCTTCTGTGATCGGT	3′
Lef1	F	5′	GCAGCTATCAACCAGATCCT	3′	Actin	F	5′	GCCTTCCTTCTTGGGTATG	3′
	R	5′	GATGTAGGCAGCTGTCATTC	3′		R	5′	ACCACCAGACAGCACTGTG	3′
Tcf4	F	5′	AATGGCCACTGCTTGATGTC	3′					
	R	5′	TACGTGATGAGAGGCGTGAG	3′					

Human									
FUZ	F	5′	GGAACTGAAGCGCTGCCTCT	3′	COL2A1	F	5′	TCTACCCCAATCCAGCAAAC	3′
	R	5′	CTGGTAGACCTCATCTTCTG	3′		R	5′	GTTGGGAGCCAGATTGTCAT	3′
SOX9	F	5′	ACACTCCTCCTCCGGCATGA	3′	ACTIN	F	5′	ACGCCAACACAGTGCTGTCT	3′
	R	5′	TTGGAGATGACGTCGCTGCT	3′		R	5′	CGATCCACACGGAGTACTTG	3′

### Expression and reporter constructs

The expression plasmid containing the cytomegalovirus (CMV) promoter linked to the mouse *Fuz* full length cDNA reverse-transcribed from total RNA of NIH-3T3 cells was constructed into pcDNA3.1 vector (Invitrogen). The 7xTopFlash reporter plasmid was constructed into luciferase vector by inserting seven *Lef/Tcf* binding sites upstream of the minimal TK promoter [94]. The mouse *Fuz* 2.4 kb promoter was constructed into the TK-luciferase vector by replacing the minimal TK promoter [95].

### Cell culture, transient transfections, luciferase and β-galactosidase assays

CHO, HEK 293 FT, LS-8 and SW1393 cells (all cells were purchased from the ATCC, except LS-8 cells [96]) were cultured in DMEM supplemented with 10% FBS and penicillin/streptomycin and transfected by electroporation. The method of transient transfections, luciferase and β-galactosidase assays were described previously [95]. LiCl was added to the specified cells at a final concentration of 10 mM, 23 h before harvest. The pcDNA3.1 empty vector was added to equalize the total amount of co-transfected expression vectors. All the plasmids were double-banded CsCl purified.

### Chromatin Immunoprecipitation (ChIP) analyses

The ChIP analyses were performed as described [97] using the ChIP Assay Kit (Upstate) with the following modifications. LS-8 cells were fed for 24 h, harvested and plated in 60 mm dishes. Cells were cross-linked with 1% formaldehyde for 10 m at 37°C the next day. Samples were incubated with rabbit anti β-catenin polyclonal antibody (Upstate 06-734) overnight at 4°C. An aliquot of the immunoprecipitated DNA (3 µl) from non-transfected cells were used for PCR (32 cycles). All reactions were done under an annealing temperature of 61°C. Two primers for amplifying the *Lef/Tcf* binding site in the *Fuz* promoter are as follows: sense- 5′- GCAACACCTTAGCACCATCA -3′ and antisense, 5′- GGCTAAATTCCTGCCTTCATC -3′. All the PCR products were evaluated on a 1% agarose gel in 1× TBE for appropriate size (201 bp) and confirmed by sequencing. As controls the primers were used without chromatin, normal mouse IgG was used replacing the β-catenin antibody to reveal non-specific immunoprecipitation of the chromatin.

### Whole-mount In Situ Hybridization

Whole-mount in situ hybridizations with digoxigenin-labelled riboprobes were performed as described [Chotteau-Lelièvre, 2006]. Expression analysis of murine genes using in situ hybridization with radioactive and non-radioactively labeled probes. In: I.A. Darby and T.D. Hewitson, Editors, Methods Mol. Biol. (third ed.), In Situ Hybridization Protocols, Humana Press, Totowa, NJ (2006), pp. 61–87.Chotteau-Lelièvre et al., 2006, using an Intavis InSitu Pro robot. The detailed robotic procedure can be found at http://empress.har.mrc.ac.uk/browser/ (gene expression section), [Chotteau-Lelièvre, 2006]. Expression analysis of murine genes using in situ hybridization with radioactive and non radioactively labeled probes. (In: I.A. Darby and T.D. Hewitson, Editors, Methods Mol. Biol. (third ed.), In Situ Hybridization Protocols, Humana Press, Totowa, NJ (2006), pp. 61–87).

### Statistics

Statistics were performed by two-sample t-test. P-values less than 0.05 were considered to be significant.
